# Supplementary material for: ZRF1 is a novel S6 kinase substrate that drives the senescence programme
Source: EMBO J. 2017 Feb 27;36(6):736–50. doi: 10.15252/embj.201694966 (PMC5350561; doi:10.15252/embj.201694966)
Supplement: Supplementary file 1 — Expanded View Figures PDF [file EMBJ-36-736-s001.pdf]

Expanded View Figures

```
Mus m.            -MLLLPSAAEGQGTAITHALTSASSVCQVEPVGRWFEAFVKRRNRNASTSFQELEDKKELSEESEDEELQLEEFPM 75
Rattus n.         -MLLLPSAAEGQGTAITHALTSASAVCQVEPVGRWFEAFVKRRNRNASTSFQELEDKKELSEESEDEELQLEEFPM 75
Homo s.           -MLLLPSAADGRGTAITHALTSASTLCQVEPVGRWFEAFVKRRNRNASASFQELEDKKELSEESEDEELQLEEFPM 75
Macaca f.         -MLLLPSPADGRGTAITHALTSASTLCRVEPVGRWFEAFVKRRNRNASASFQELEDKKELSEESEDEELQLEEFAM 75
Bos t.            -MLLLPSAADGQGTAITHALTSASTLCQVEPVGRWFEAFVKRRNRNASASFQELEDKKELSEESEDEELQLEEFPM 75
Gallus g.         ---MLRGAHEGQLTAVTGALTAASVLCQVEPVGRWFEAFIKRRNINVSASFQELEDEKELSEESGDEELQLEEFPM 73
Xenopus t.        ---MLIEAQAEQCTFVTRGLCAAAVLCQVEPVGRWFEAFIKRRNRNVSASFQELEDEKESSEESEDEEFQLEEFPL 73
Danio r.          ---MLTEALEGQLTVVYN-TVAASVQVQVEPVGRWFEAFLKRRNRNVSASFQELEEEEELSEEEEDEELQLEEYPM 72
Saccharomyces c.  MFSLPTLTSDITVEVNSSATKTPFVRRPVEPVGKFFLQHAQRTLRNHTWSEFERIEAEKNVKTVDESNVDPDELLF 78
                     :   .             :.      *****::*  . :*   * : *  *  :  ::  :   :.:.: :*  :
```

Figure EV1. Sequence homology of ZRF1 phosphorylation motif across species.
Alignment of amino acid sequences of ZRF1 from different species encompassing the S6K consensus phosphorylation site motif (R/K-X-R/K-X-X-S/T) underlined; the arrow indicates the phosphorylation site.

 © 2017 The Authors

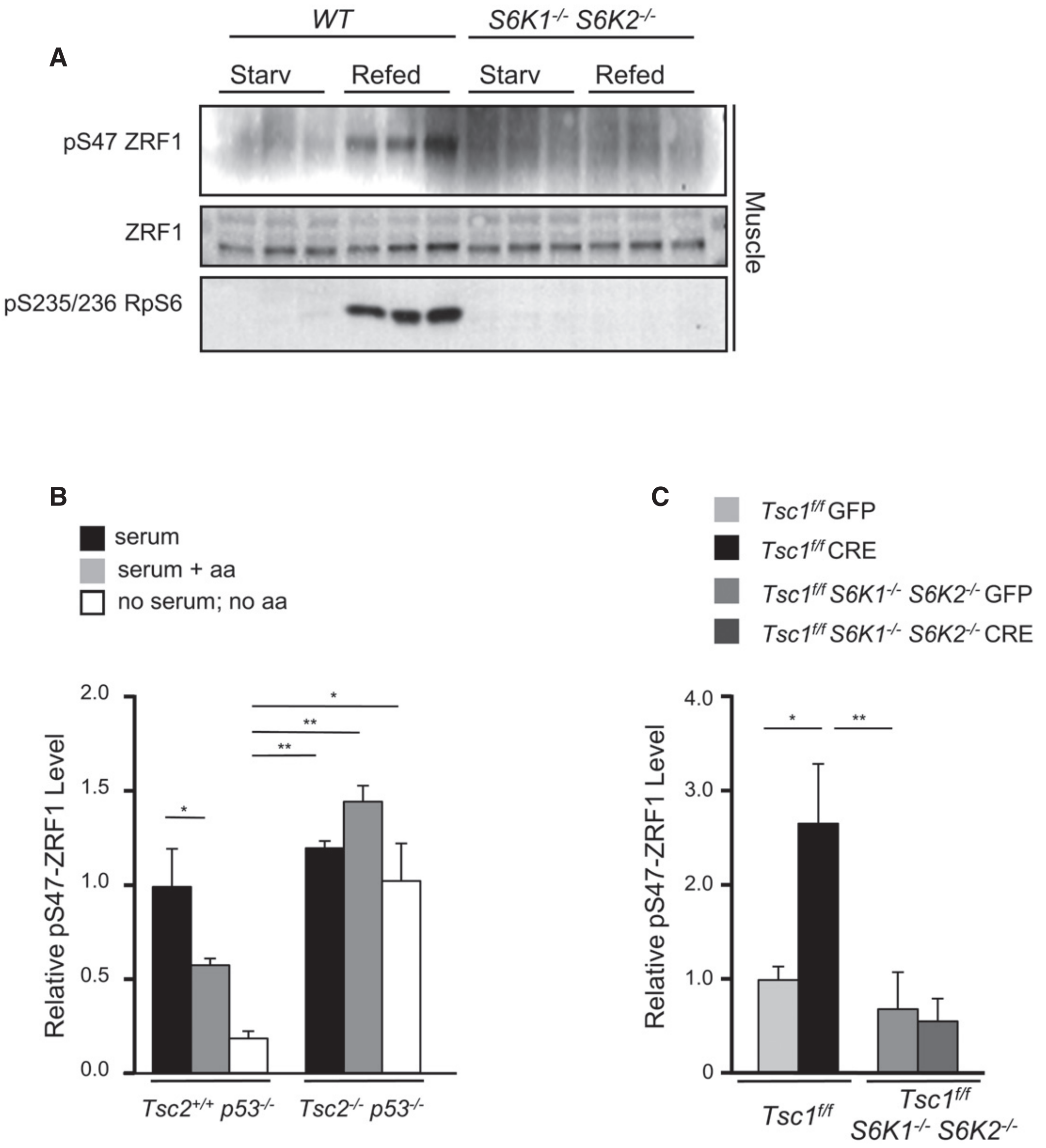

Figure EV2. ZRF1 phosphorylation in tissue and cultured cells.

A Representative immunoblot analysis of proteins extracted from skeletal muscles of WT and *S6K1*$^{-/-}$*S6K2*$^{-/-}$ mice that were starved overnight or starved overnight and refed for 4 h (n = 3 mice for each genotype).

B, C Densitometric analyses of the blots in Fig 6D and E, respectively. The phosphorylation level of ZRF1 is normalized to tubulin or actin protein level and densitometric results are presented as fold changes relative to the control. Data are reported as mean ± SEM of n = 3; *P < 0.05, **P < 0.01, ANOVA multiple comparisons.

© 2017 The Authors
